# Case Report: Migraine-Induced Dystonia of the Lower Extremities

**DOI:** 10.3389/fneur.2022.855698

**Published:** 2022-05-09

**Authors:** Ting Jiang, Yinyin Xie, Buajieerguli Maimaiti, Yu Cheng, Zhaoran Li, Hongmei Meng

**Affiliations:** ^1^Department of Neurology, The First Hospital of Jilin University, Changchun, China; ^2^Department of Neurology, The First Affiliated Hospital of Zhengzhou University, Zhengzhou, China; ^3^Academy of Medical Sciences, Zhengzhou University, Zhengzhou, China

**Keywords:** migraine, dystonia, movement disorder, patent foramen ovale, case report

## Abstract

Migraine is a highly prevalent neurological disorder characterized by recurrent, unilateral, or bilateral throbbing severe headaches. Currently, there are extremely rare cases of migraine-induced dystonia. A 52-year-old woman was admitted for intractable migraine for about 5 days and walking difficulties for 1 day. The symptom of an inability to walk appeared on the fourth day of the headache attack lasting for 1 day and resolved on its own as the headache subsided. The same symptoms appeared once 6 years ago. Neurological examination, brain Magnetic resonance imaging (MRI), laboratory tests of blood and cerebrospinal fluid (CSF) were normal. The contrast transcranial Doppler echocardiography (cTCD) revealed a latent and massive right-to-left shunt (RLS) after the release of the Valsalva maneuver. The patient was diagnosed with migraine-induced dystonia of the lower limbs. Oral ibuprofen and flunarizine and avoidance of increased chest pressure maneuvers were used for treatment and prevention. During the 6-month follow-up, the patient was free of headaches and walking difficulties. Our study reported a rare case of migraine-induced dystonia of the lower extremities.

## Introduction

Migraine is the second most common neurological disease worldwide, with an annual incidence of up to 15% in the general population (1). Migraine is frequently characterized by recurrent, unilateral, or bilateral throbbing, severe headache. Dystonia is a movement disorder manifesting as abnormal movements or postures, or both, caused by continuous or intermittent muscle contractions, and the movements and postures are frequently repetitive with an annual incidence of 15–30 per 100,000 in the general population (2). There have been some reports of patients suffering from migraine and dystonia simultaneously (3, 4). However, reports of migraine-induced dystonia are particularly rare, and the mechanisms involved are unclear so far (5).

Here, we report a rare case of a patient with migraine-induced dystonia of the lower extremities manifested by the inability to walk. The dystonia symptoms resolved spontaneously with migraine resolution. Auxiliary examination revealed that she had a patent foramen ovale and a potentially large right-to-left shunt. The same symptoms appeared twice in 6 years. We also make reasonable guesses about possible etiologies.

## Case Description

### History

A 52-year-old woman without a family history of migraine and dystonia was admitted to our department due to intractable migraine for about 5 days and walking difficulties for 1 day. She has had a history of headaches for over 20 years with one or two attacks per year described as a throbbing or swelling pain in the occipital region or even the whole brain with an intensity of 6 to 9/10. There was no significant aura before the attack. The headache attack was accompanied by numbness and coldness of the painful area and stiffness of the neck, and nausea and vomiting without photophobia or phonophobia. At worst, she could not take in any food or medicine because of vomiting. The headache usually lasted for 5–6 days and could last up to 15 days, and was slightly relieved by ibuprofen and flunarizine. On the fourth day of this attack, she suddenly suffered an inability to walk lasting for 1 day. She could stand on her own without any dizziness. However, every time she tried to start walking, she felt stiffness in both lower extremities and was unable to step and walk, but could only maintain an upright position. And, it did not change with prolonged standing time. In addition, no movement other than walking was affected. When she was lying down, the movement of both lower extremities was not restricted and she could raise and lower her legs and move them in any direction at will. The same symptom happened once 6 years ago, and it resolved on its own after lasting for 1 h.

### Examinations and Imaging Findings

**Figure 1:**
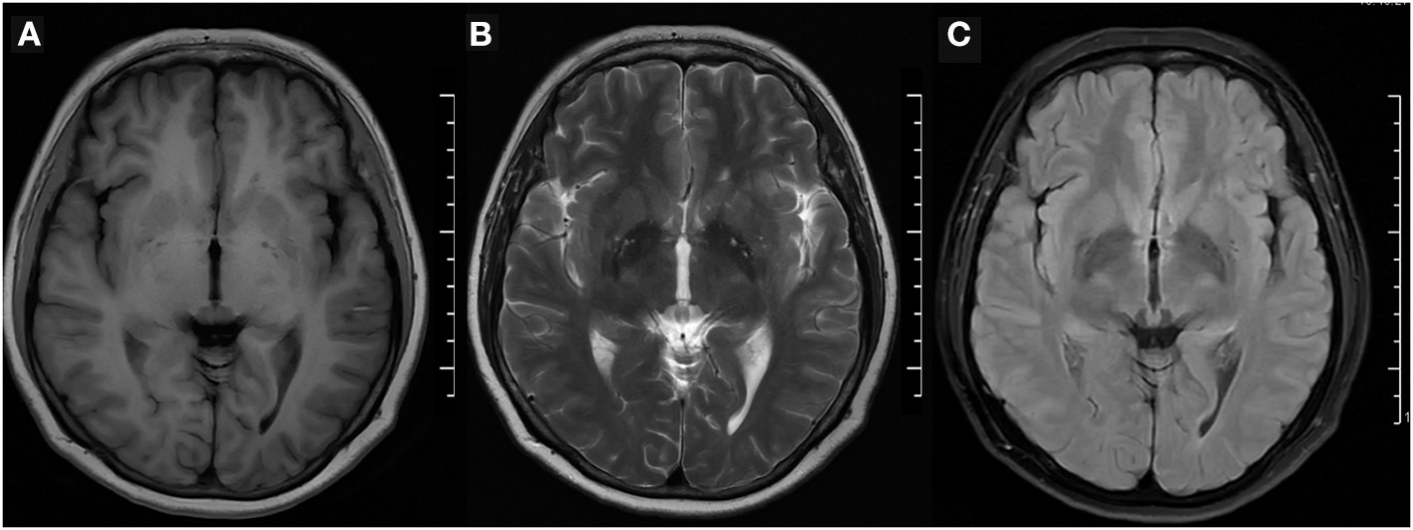
Brain MRI. **(A)** T1-weighted, **(B)** T2-weighted, and **(C)** fluid-attenuated inversion recovery (FLAIR) imaging were normal. MRI, magnetic resonance imaging.

**Figure 2:**
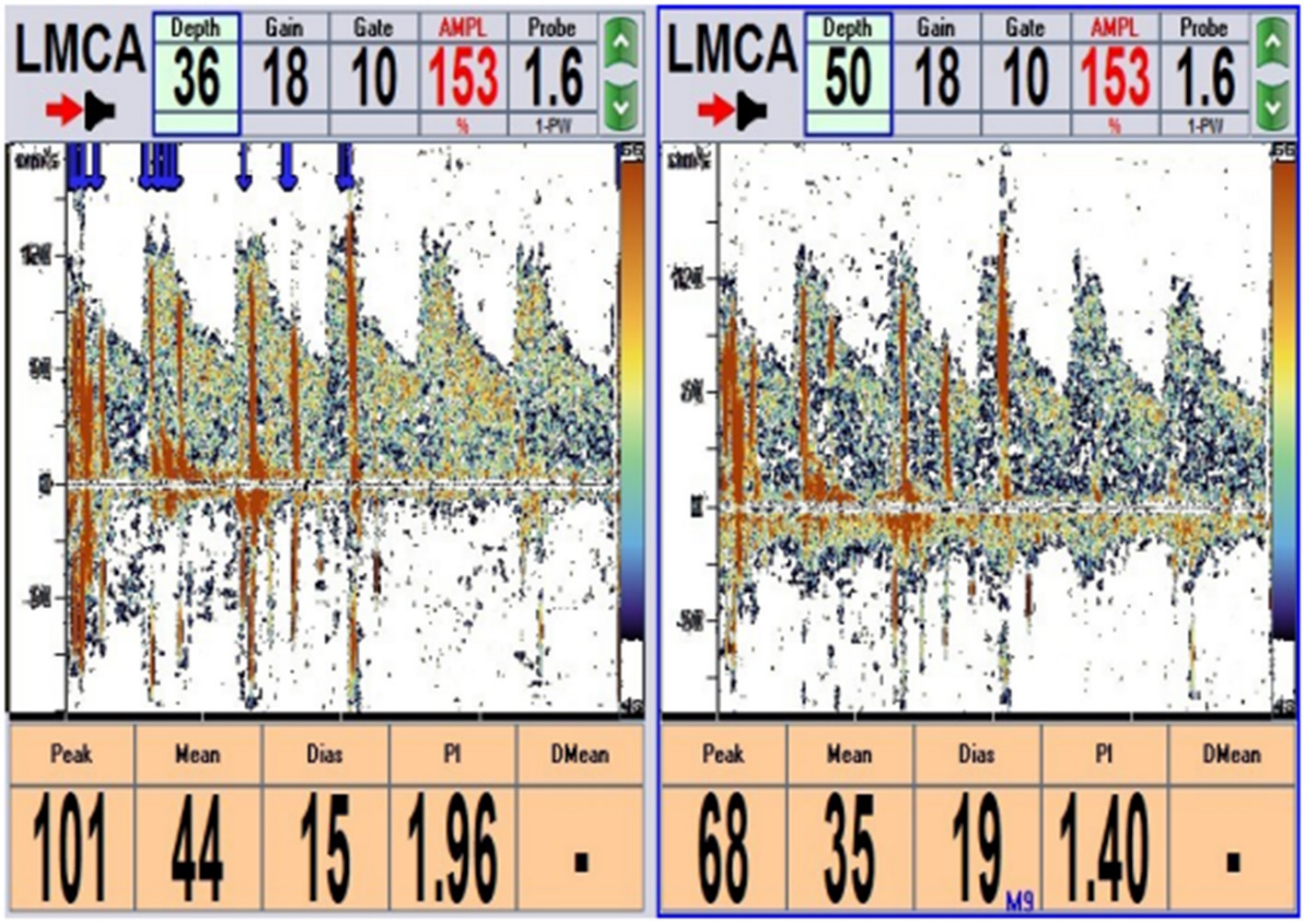
cTCD: More than 25 microbubbles were detected after 2 s of Valsalva maneuver detecting from the left middle cerebral artery (LMCA) (single-channel and double depth) (blue arrow). cTCD, contrast transcranial Doppler echocardiography.

The physical examination and vital signs were unremarkable, and the neurologic examination was normal. The patient showed a task-specific lower limbs dystonia, characterized by the appearance of sustained dystonic extension of both knees induced by stepping or walking attempts. Magnetic resonance imaging (MRI) scan of the brain showed no significant abnormalities (Figure 1). The patient underwent a lumbar puncture, the cerebrospinal fluid (CSF) pressure was 125 mm H_2_O (normal range: 80–180 mmH_2_O), and the biochemistry and cytology of the CSF were negative. Her blood tests were regular, including routine blood work, liver function, kidney function, ions, D-dimer, and markers of myocardial damage. The contrast transcranial doppler echocardiography (cTCD) showed a latent and massive right-to-left shunt (RLS), that was, more than 25 microbubbles were detected using insonation of the left middle cerebral artery (LMCA) after the release of the Valsalva maneuver (Figure 2). No microbubble was seen in the resting state. Right heart contrast echocardiography confirmed the result. Her echocardiography revealed no abnormalities in the structure and function of the heart at rest.

### Diagnosis and Treatment

Finally, the patient was diagnosed with migraine and dystonia of the lower extremities induced by it. The cTCD and right heart contrast echocardiography suggested that she had a patent foramen ovale (PFO). Transesophageal echocardiography (TEE) was required to clarify her heart condition further. The patient's headache lasted for 12 days and was relieved with oral ibuprofen (two capsules per day) and flunarizine (10 mg per day). The symptom of the inability to walk cleared spontaneously staying for 1 day, and she was capable of walking slowly and awkwardly during the consultation. Other than medicines for headaches, no additional treatment was given, similar to the situations 6 years ago. Considering the presence of PFO, we recommended she avoid actions that would increase chest pressure, such as diving, violent coughing, and strenuous exercise, which would be of considerable benefit for preventing her headaches. At a follow-up 6 months after the attack, the patient had no episodes of headache and walking difficulty. Unfortunately, owing to financial problems, the patient did not undergo a relevant genetic test, making it difficult to explore the etiology of her condition further.

## Discussion

In this case, we demonstrate a patient presenting with migraine combined with dystonia triggered by it, with the dystonia manifesting as a walking impediment. Migraine contains three main types: migraine with aura, migraine without aura, and chronic migraine. There is a strong association between migraine and PFO. The prevalence of PFO accounts for 46.3–88% of migraineurs with aura and 16.2–34.9% of migraineurs without aura (6). Recent studies suggest that PFO may trigger migraine and is positively correlated with attack frequency but are not significantly related to attack symptoms (7). Previous studies have confirmed that suffering from migraine can affect patients' gait and balance. Akdal et al. (8) discovered that migraineurs without manifesting vertigo had more incredible sway velocity when standing, more significant offset center of gravity (COG) alignment, wider step width, and slower speed. Machado Maciel et al. (9) found migraineurs experienced longer step width with increasing light and sound levels. However, in our case, the patient's twice walking disability within 6 years both occurred during the attack of migraine without dizziness and balance impairment and with normal motor and sensory examinations at rest, suggesting that the symptom was migraine-induced-dystonia of lower extremities.

The classification of dystonia is based on two main clinical features: the age of onset, distribution of symptoms, concomitant symptoms, and etiology (2). Combining the patient's medical history, we considered the patient's dystonia of the lower extremities as a task-specific walking dystonia. No significant geste antagoniste or sensory trick was found in the patient's description and examination. Kemp et al. (10) previously reported a case of delayed dystonia of the left leg secondary to traumatic brain injury, with some resemblance to our case. This patient presented with a persistent extension of the left knee and was task-specific, appearing only when he walked forward. Previous studies have revealed that focal task-specific lower extremity dystonia is associated with prolonged vigorous repetitive activity. Katz et al. (11) reported seven patients with task-specific lower extremity dystonia who had multiple exercise triggers. Their motor triggers included prolonged bicycling, hiking, long-distance running, and drumming. In addition, task-specific dystonia usually progresses continuously. This reminds us of the patient in this case, who has been persistently walking briskly 20,000 steps per day, about 14 km per week, for more than a decade. The duration of her two migraine-induced dystonic episodes that occurred within 6 years became longer, from an hour to a day, although the symptoms were the same on both occasions. Dystonia was elicited by walking attempt, in a task-specific manner. However, we found no significant geste antagoniste or sensory tricks, which may need to be detected in her subsequent episodes and long-term follow-up.

During the diagnosis of walking disability in the patient, we are not inclined to consider functional movement disorders or extrapyramidal symptoms caused by medication side effects as a diagnosis. Patients with functional movement disorders are often associated with anxiety and depression, and a higher proportion of patients have suffered psychological trauma (12, 13). The patient in this case had not experienced significant previous trauma, and her Hamilton Anxiety and Depression Scale test results did not indicate a tendency to suffer from anxiety or depression. Since the patient was capable of walking when she arrived at the hospital, we did not perform suggestive therapy on her. Studies have found that long-term oral administration of flunarizine significantly increases the risk of movement disorders in a dose-dependent manner, including dystonia, parkinsonism, akathisia, tremor, and tardive dyskinesia (14). The patient in our case did not take flunarizine to prevent migraine. She usually took ibuprofen (two capsules per day) and flunarizine (10 mg per day) orally to relieve her headache when it occurred. Furthermore, due to severe nausea and vomiting and even inability to take her medication during the first 2–3 days of the migraine attack, she usually took oral flunarizine for no more than 5 days during the course. Actually, ibuprofen was the more effective and more often chosen medication for her in most conditions. In addition, the patient's presentation differs from that of paroxysmal kinedigenic dystonia (PKD). PKD has a usual episode duration of <1 min, and the reported presentations of PKD do not cover walking disorders like this patient's (15). As a result, combining the patient's medical history and clinical manifestations, the final diagnosis was migraine-induced dystonia of the lower extremities.

Prior researches have reported several patients with migraine combined with dystonia and the relevant genetic mutation in these patients. Dale et al. (3) and Cuenca-León et al. (16) reported patients with benign paroxysmal torticollis (BPT) and hemiplegic migraine (HM) accompanied by *PRRT2* and *CACNA1A* gene mutation respectively. Gardiner et al. (17) reported individuals with PKD and migraine accompanied by *PRRT2* gene mutation. Weber et al. (4) and Gardiner et al. reported patients with paroxysmal exertion-induced dyskinesia (PED) and migraine accompanied by *GLUT1* and *SLC2A1* gene mutation respectively. In these cases, the dystonia usually manifests as abnormal movements, and there was no specific association between migraine and dystonia episodes. To our knowledge, only one study mentioned a patient with PED caused by migraine whose dystonia symptoms presented as face contraction with dysarthria, preceded the onset of migraine, and resolved with the relief of migraine (5). In our case, there was a definite association between the patient's two episodes of dystonia and migraine attacks, which occurred in the duration of migraine and disappeared with the reduction of the headache. Moreover, the dystonia presented as walking difficulties of the lower extremities, which was not previously documented.

The specific mechanisms of migraine and dystonia are not well identified so far. Cortical spreading depression (CSD) plays a vital role in the pathophysiology of both migraine and task-specific dystonia (18, 19). However, CDS is not specific and may also be an accompanying phenomenon in the disease process (20). Previously reported channel genes associated with migraine include *CACNA1A, ATP1A2, ATP1A3, ATP1A4, SCN1A, PRRT2, PNKD, SLC2A1, SLC1A3* and *SLC4A4* (21, 22). Different types of myotonia correspond to different pathogenic genes, such as the TOR1A gene mutation in DYT1, the *TUBB4A* gene mutation in DYT4, and the *GNAL* gene mutation in DYT25 (2). Among them, channel genes associated with paroxysmal dystonia include *PRRT2, PNKD, ATP1A3, SLC2A1*, and *SCN8A* (23). It is obvious that migraine and dystonia possess some genetic regulatory mechanisms in common. Given the mutation concerning genes, channel impairment may be an intrinsic mechanism commonly shared by both (24). Gene mutation results in disruption of neurotransmitter release, which in turn impairs synaptic release. The *ATP1A2* and *ATP1A3* genes, which are different isoforms encoding the Na+/K+-ATPase (NKA) alpha subunit, are associated with FHM2 (*ATP1A2*), childhood alternating hemiplegia (*ATP1A2/A3*), RDP (*ATP1A3*), cerebellar ataxia-reflex loss-progressive optic atrophy (*ATP1A3*), and recurrent encephalopathy with cerebellar ataxia (*ATP1A3*), respectively (25). The *ATP1A4* mutation is a novel gene mutation that was detected associated with FHM (22). *PRRT2* mutations lead to dysregulation of transmembrane calcium and sodium channels, resulting in diseases such as FHM2 and PKD (17). Mutations in *SCN1A*, which encodes the pore-forming a1 subunit of the neuronal voltage-gated sodium channel Nav1.1, are likely to cause neurological disorders such as FHM and HM in patients (21). Mutations in *PNKD* disrupt neurotransmitter regulation, which in turn is implicated in HM and PNKD (26). The possibility of phenotypic pleiotropy of genes associated with migraine and dystonia (Table 1), meanwhile, cannot be excluded. However, the significant temporal association between migraine and dystonia and the uniqueness of the dystonia manifestation in this case should still be acknowledged.

**Table 1:** Main phenotypic pleiotropy in genes associated with migraine and dystonia.

**Genes**	**Migraine**	**Dystonia**	**Other phenotypes**
*KCNA1* (OMIM * 176260)	Migraine	Myokymia syndrome	Episodic ataxia-1 (EA1) Epilepsy
*CACNA1A* (OMIM * 601011)	Familiar hemiplegic migraine-1 (FHM1)	Benign paroxysmal torticollis (BPT) Blepharospasm (BSP)	Episodic ataxia-2 (EA2) Spinocerebellar ataxia Epilepsy Epilepsic encephalopathy
*ATP1A3* (OMIM * 182350)	Hemiplegic migraine (HM)	Rapid-onset dystonia-parkinsonism (RDP) (Dystonia-12)	Alternating hemiplegia of childhood CAPOS syndrome Developmental and epileptic encephalopathy
*PRRT2* (OMIM * 614386)	Familiar hemiplegic migraine-2 (FHM2)	Benign paroxysmal torticollis (BPT) Paroxysmal kinedigenic dystonia (PKD)	Episodic ataxia (EA) Epilepsy Epilepsic encephalopathy
*SLC2A1* (OMIM * 138140)	Hemiplegic migraine (HM)	Paroxysmal exertion-induced dyskinesia (PED) Paroxysmal nonkinesigenic dyskinesia (PNKD)	GLUT1 deficiency syndrome (GLUT1DS) Epilepsy Epilepsic encephalopathy
*SCN1A* (OMIM * 182389)	Familiar hemiplegic migraine-3 (FHM3)	Dystonia	Dravet syndrome Febrile seizures, familial, 3A Generalized epilepsy with febrile seizures plus, type 2 Developmental and epileptic encephalopathy 6B, non-Dravet
*PNKD* (OMIM * 609023)	Hemiplegic migraine (HM)	Paroxysmal nonkinesigenic dyskinesia (PNKD)	–
*ATP1A2* (OMIM * 182340)	Familiar hemiplegic migraine-2 (FHM2) Familial basilar	–	Alternating hemiplegia of childhood Developmental and epileptic encephalopathy Fetal akinesia, respiratory insufficiency, microcephaly, polymicrogyria, and dysmorphic facies
*ATP1A4* (OMIM * 607321)	Familiar hemiplegic migraine (FHM)	–	–
*SCN8A* (OMIM * 600702)	–	Myoclonus, familial, 2	Cognitive impairment with or without cerebellar ataxia Developmental and epileptic encephalopathy Seizures, benign familial infantile

Management of migraine-induced dystonia is primarily focused on migraine prevention and treatment. Current randomized controlled studies revealed an unremarkable effect of PFO closure on migraineurs (27). A further genetic test is required, even though it has a limited impact on the patient's treatment, it may play a profound role in expanding our understanding of the underlying mechanisms of migraine and dystonia. Further follow-up is required as well.

## Conclusion

In conclusion, our study suggests a rare case of migraine-induced dystonia of the lower extremities, which broadens our insight into migraine and dystonia. To date, only one case has been reported previously, and there are some discrepancies with our case. Further fundamental analyses are needed to explore the potential mechanisms.

## Data Availability Statement

The original contributions presented in the study are included in the article/supplementary material, further inquiries can be directed to the corresponding author/s.

## Ethics Statement

Written informed consent was obtained from the individual(s) for the publication of any potentially identifiable images or data included in this article.

## Author Contributions

TJ and HM carried out the patient information acquisition and manuscript preparation and drafted the manuscript and prepared the figure and table. YX and BM checked the literature and developed the idea of the study. YC and ZL revised the manuscript and gave the final approval. HM contributed to the conception and design of the manuscript. All authors read the approved and final manuscript.

## Funding

This study was supported by grants by the National Natural Science Foundation of China (No. 81871008).

## Conflict of Interest

The authors declare that the research was conducted in the absence of any commercial or financial relationships that could be construed as a potential conflict of interest.

## Publisher's Note

All claims expressed in this article are solely those of the authors and do not necessarily represent those of their affiliated organizations, or those of the publisher, the editors and the reviewers. Any product that may be evaluated in this article, or claim that may be made by its manufacturer, is not guaranteed or endorsed by the publisher.
